# Detection of hypoplastic left heart syndrome anatomy from cardiovascular magnetic resonance images using machine learning

**DOI:** 10.1007/s10334-023-01124-9

**Published:** 2024-01-12

**Authors:** Dominik Daniel Gabbert, Lennart Petersen, Abigail Burleigh, Simona Boroni Grazioli, Sylvia Krupickova, Reinhard Koch, Anselm Sebastian Uebing, Monty Santarossa, Inga Voges

**Affiliations:** 1 Department of Congenital Heart Disease and Pediatric Cardiology, DZHK (German Center for Cardiovascular Research), Partner Site Hamburg/Kiel/Lübeck, University Hospital Schleswig-Holstein, Kiel, Germany; 2 Department of Computer Science, Kiel University, Kiel, Germany; 3 Department of Pediatric Cardiology, Royal Brompton Hospital, London, UK

**Keywords:** Congenital heart disease, Convolutional neural network, Classification, Anatomy

## Abstract

**Objective:**

The prospect of being able to gain relevant information from cardiovascular magnetic resonance (CMR) image analysis automatically opens up new potential to assist the evaluating physician. For machine-learning-based classification of complex congenital heart disease, only few studies have used CMR.

**Materials and methods:**

This study presents a tailor-made neural network architecture for detection of 7 distinctive anatomic landmarks in CMR images of patients with hypoplastic left heart syndrome (HLHS) in Fontan circulation or healthy controls and demonstrates the potential of the spatial arrangement of the landmarks to identify HLHS. The method was applied to the axial SSFP CMR scans of 46 patients with HLHS and 33 healthy controls.

**Results:**

The displacement between predicted and annotated landmark had a standard deviation of 8–17 mm and was larger than the interobserver variability by a factor of 1.1–2.0. A high overall classification accuracy of 98.7% was achieved.

**Discussion:**

Decoupling the identification of clinically meaningful anatomic landmarks from the actual classification improved transparency of classification results. Information from such automated analysis could be used to quickly jump to anatomic positions and guide the physician more efficiently through the analysis depending on the detected condition, which may ultimately improve work flow and save analysis time.

## Introduction

In recent years, the great potential of artificial intelligence to support clinical evaluation of cardiovascular magnetic resonance imaging (CMR) data has become increasingly apparent [1, 2]. Several CMR studies applied machine learning to congenital heart disease to address specific tasks related to segmentation [3], assessment of surgical results [4], prediction of prognosis [5, 6], hierarchical clustering of congenital heart disease [7] and data augmentation [3, 8]. Machine learning classification of complex congenital heart disease has been demonstrated using echocardiography [9], but only few studies exist that have used CMR for classification of complex congenital heart disease using machine learning [7].

In this study, a new approach of machine learning for CMR diagnostic classification of patients with hypoplastic left heart syndrome (HLHS) is presented. HLHS is one of the most severe forms of congenital heart disease and is characterised by hypoplasia of the left ventricle, stenosis or atresia of the mitral valve and aortic valve as well as hypoplasia of the ascending aorta [10]. At many cardiac centres, the Norwood operation is the surgical palliation of choice followed by creation of an upper cavopulmonary connexion and completion of the Fontan circulation with a total cavopulmonary connexion (TCPC) [11]. Due to the underdeveloped left side of the heart as well as the surgical interventions, the anatomy differs greatly from healthy individuals and seems suitable for classification based on the geometric arrangement of anatomical landmarks.

This study aims to evaluate and optimise the use of a deep convolutional neural network (CNN) for detection of anatomical landmarks in the cardiovascular system. Furthermore, it was the goal to quantify the impact of each anatomical landmark on the classification system and to assess how the arrangement of landmarks can be standardised amongst datasets. The approach was applied to patients with hypoplastic left heart syndrome (HLHS) in Fontan circulation and healthy controls.

## Materials and methods

### Study participants and CMR data acquisition

The study was approved by the local ethics committee. Informed consent was provided by all subjects or by their legal guardians. 46 patients with HLHS with a median age of 9.7 years (y) (range 2.9–22.5 y), studied by CMR at a median of 7.2 y (range 0.9–21.4 y) after TCPC completion with an intra-atrial lateral tunnel, as well as 33 healthy controls (median age at CMR 13.8 y, range 8.6–17. 5 y) were retrospectively included. CMR examinations included a stack of axial cine images with coverage of the entire chest using an ECG-gated Steady-State Free Precession (SSFP) sequence. The resolution of the scans varied and the heart was not uniformly centred. The scanning parameters were as follows: 17–54 slices, slice thickness 5–8 mm, in-plane-resolution 1.2 × 1.2–2.3 × 2.3 mm and an acquisition matrix of 132 × 192–192 × 256 pixels.

### Method overview

**Fig. 1:**
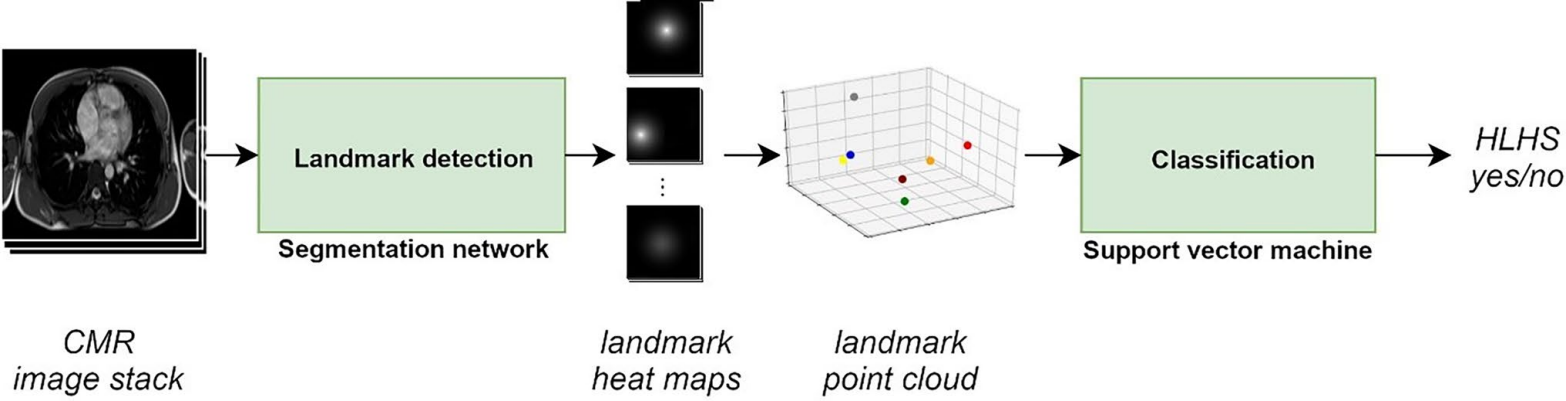
Classification pipeline: A segmentation network detects anatomical landmarks in an axial cine stack. Arranged as a point cloud, the landmarks enter a support vector machine (SVM) for classification of the landmark arrangement in HLHS patients and controls with normal cardiovascular anatomies

Cardiovascular anatomy was classified by the spatial arrangement of distinctive anatomical landmarks. The detection of anatomical landmarks was based on axial cine images and accomplished using a tailor-made deep convolutional neural network (CNN) for segmentation. The actual classification was based exclusively on the coordinates of the detected landmarks and performed by a linear support vector machine (SVM). A flow chart of the method is illustrated in Fig. 1.

The neural network implementation was based on Tensorflow (version 2.8.0) using the Keras API [12]. The support vector machine was implemented using the Scikit-learn software library (version 1.0.2).

### Splitting of data

For training and evaluation purposes of the CNN, cross-validation was performed with 5-fold. Each fold was composed of 63–64 (~ 80%) train and 15–16 (~ 20%) validation datasets, which were sampled evenly from patients and controls ensuring that the training and the validation datasets of each fold contain an equal ratio of patients and controls. Each patient or control case was included in exactly one of the 5 validation datasets.

For each fold, the CNN segmentation network and the SVM classifier were trained with randomly initiated weights on the training data. Evaluation was performed for each fold on the validation data. Evaluation scores were averaged over all folds.

The sample sizes in this study appeared too small for separating a test set of significant size. We followed the common practice to prevent overfitting by cross-validation on fixed hyper-parameters [13].

### Data pre-processing

CMR datasets were cropped at slice number 33 which was at the lower end of the image stack beyond the diaphragm for all datasets. None of the annotated landmarks were located outside the cropped image stack. For datasets with less than 33 slices, extra slices with black content were appended to complete a number of 33 slices. A resampling of slice images to a 256 × 256 resolution was performed. Overall, data pre-processing resulted in an image stack of size 33 × 256 × 256.

### Data augmentation

#### CNN

Augmentation of data was tested by adding each data set another time with modifications applied using the image augmentation Python library Albumentations (https://albumentations.ai, version 1.3.0). Parameters for random shift, rotation and scaling were: shift_limit = 0.02, rotation_limit = 4, scale_limit = 0.15, *p* = 1. Parameters for blurring and optical distortion were: blur_limit = 4 and distort_limit = 3, *p* = 1.

#### SVM

For training of the SVM, augmentation of datasets was tested by including each set of landmarks again 400 times, with positions of the landmarks shifted by uniformly distributed random values between -10 and 10 mm in each spatial direction.

#### Landmark definition

**Fig. 2:**
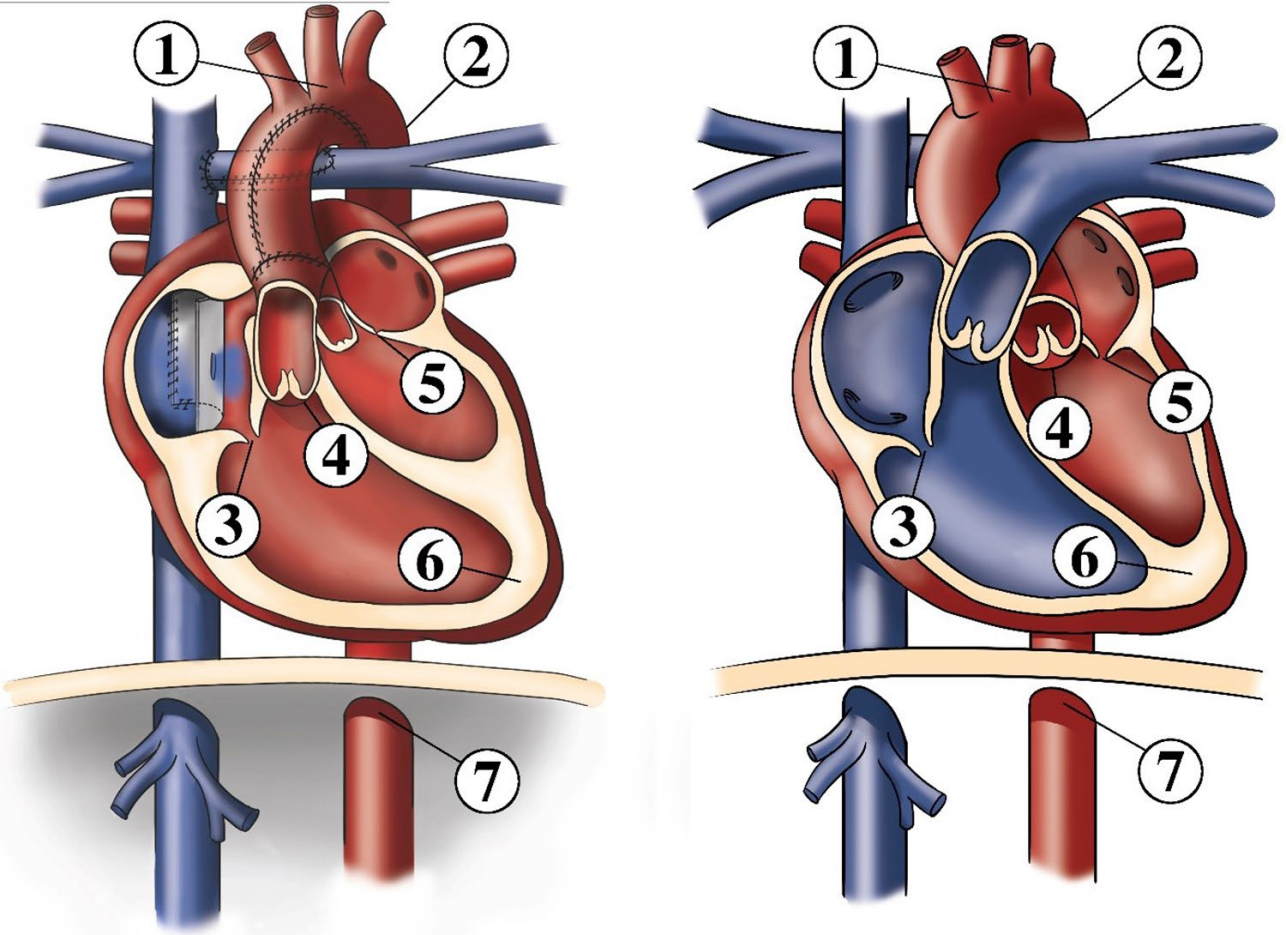
Comparison of the cardiovascular anatomy of HLHS in Fontan circulation with intra-atrial tunnel (left) and the normal cardiovascular anatomy (right). Seven landmarks were used for classification: (1) origin of the left common carotid artery, (2) aortic isthmus, (3) tricuspid valve, (4) neo-aortic valve in HLHS patients, aortic valve in healthy controls, (5) mitral valve, (6) right ventricular apex, (7) descending aorta at diaphragm level

**Fig. 3:**
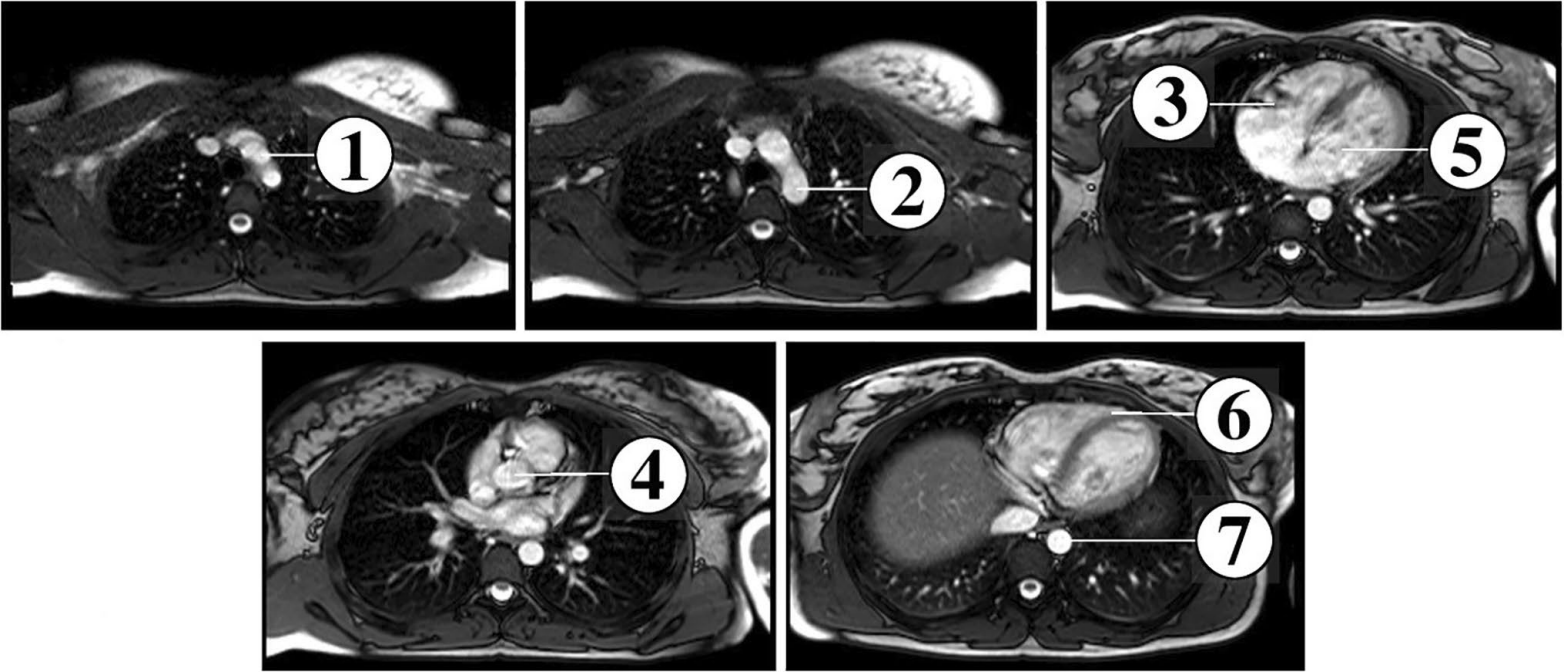
Landmark positions in axial slices. For definition of landmarks, see Fig. 2

Seven anatomical landmarks were used in this study (Figs. 2, 3).

The landmarks used in this study met three criteria. First, the landmarks used could be defined for HLHS patients as well as healthy individuals. Second, with the inclusion of landmarks in vessels, heart valves, and cardiac apex, they represented the spatial arrangement of all important cardiac structures. And third, the landmarks used could be recognised in MRI images with sufficient image quality. However, small structures such as the landmark for the origin of the left common carotid artery were expected to have less uncertainty in defining the landmark position than other structures such as the right ventricular apex for example. Because the native pulmonary valve in HLHS patients in Fontan circulation functions as neo-aortic valve and has already been functionally assigned to the landmark for the aortic/neo-aortic valve, detection of the pulmonary valve by a separate landmark was omitted. The origin of the left common carotid artery (landmark 1) was defined as the point in the centre of the left common carotid artery in the first slice superior to the aortic arch. The aortic isthmus (landmark 2) was defined as the centre point in the aortic constriction distal to the origin of the left subclavian artery. Tricuspid valve (landmark 3), neo-aortic/aortic valve (landmark 4), and mitral valve (landmark 5) were defined as the centre points in the corresponding valve annuli. The right ventricular apex (landmark 6) was defined in the first slice in the myocardium centred just below the right ventricular cavity. The landmark in the descending aorta (landmark 7) was defined in the centre of the descending aorta at the level of the diaphragm.

### Manual landmark annotation and evaluation

Manual annotation served as ground truth for the CNN training and was used for evaluation of predicted results. Two observers prepared annotations of landmarks in the end-diastole of CMR data for patients and healthy controls. Observer 1 was an experienced cardiologist and observer 2 was a trained medical student. A landmark annotation consisted of the selection of a voxel position in the axial cine stack. Annotations of the first observer served as ground truth for neural network training. For each predicted landmark, we assessed average displacement and standard deviation between predicted and annotated landmarks as well as the interobserver variability for healthy controls, patients and the combined group of both. Annotation data were prepared using cvi42 (Circle Cardiovascular Imaging Inc., version 5.13) and were exported as xml files.

### Landmark detection

Landmark detection by the neural network was realised through a heatmap regression such that each landmark has a separate volumetric output containing a heatmap centred at the landmark position [14–16].

**Fig. 4:**
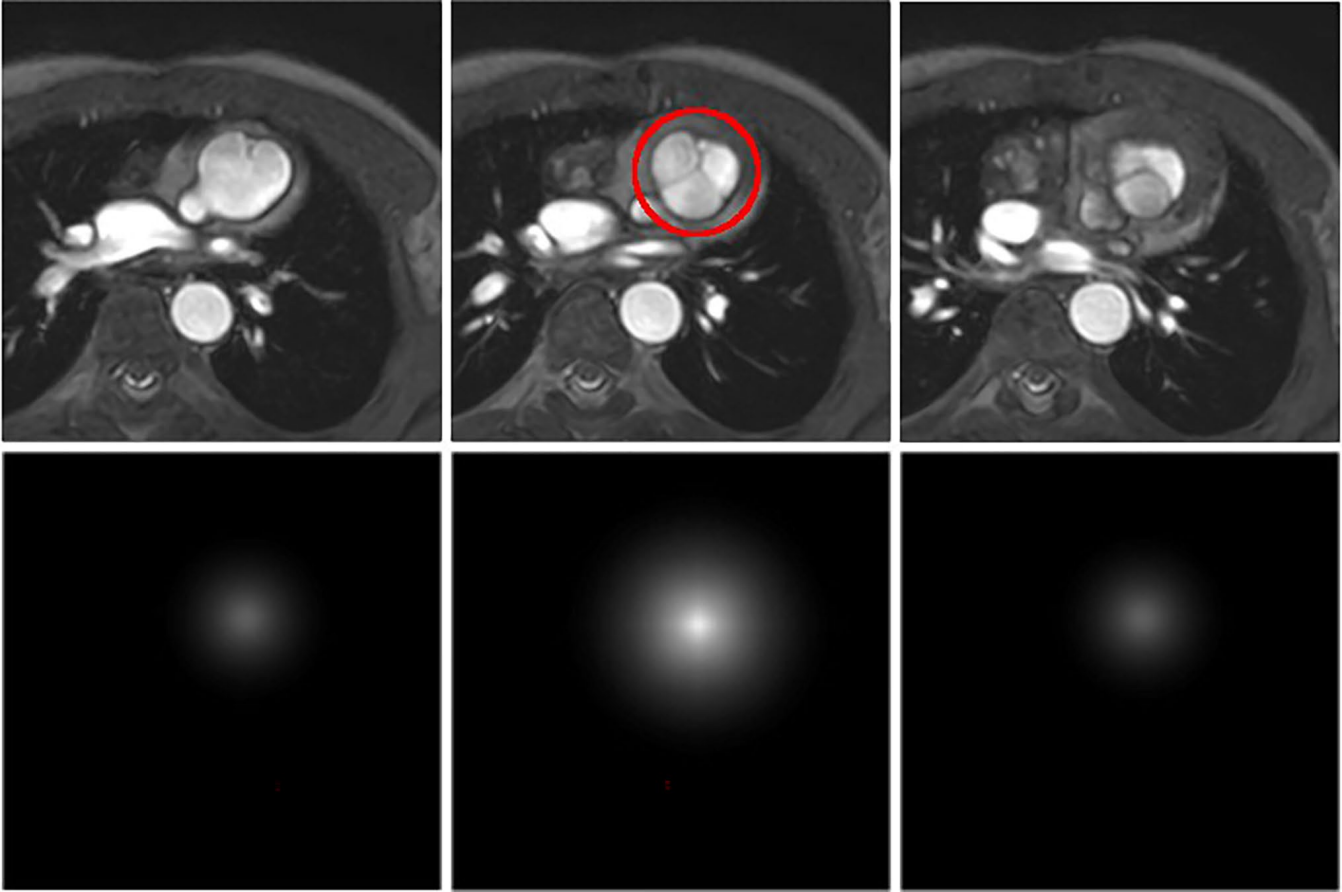
Position of the neo-aortic valve landmark (red circle) in an 8-year-old HLHS patient (top row) and the corresponding heat map (bottom row). Three adjacent slices are shown from left to right

Landmark annotations were converted into 3D heatmaps before transferring them to the CNN, Fig. 4, and accordingly, the network predicted heatmaps. The heatmaps had the shape of a Gaussian distribution with maximum values *k* at the landmark positions and decreasing values with increasing distance of the voxel *v* to the landmark positions *p*_*i*_ according to

1 $$t_{i} = ke^{{\frac{{ - \left( {v - p_{i} } \right)^{2} }}{{2\sigma^{2} }}}}$$

where *t*_*i*_ is the temperature of the *i* th heat map we regress against for landmark *i*, σ is the standard deviation of the distribution. The standard deviation σ was chosen to be the voxel size in each direction of space. In order to improve training stability especially in the early epochs, a high parameter *k* = 10^3^ was chosen in accordance with recommendations [16].

### Segmentation model architecture

**Fig. 5:**
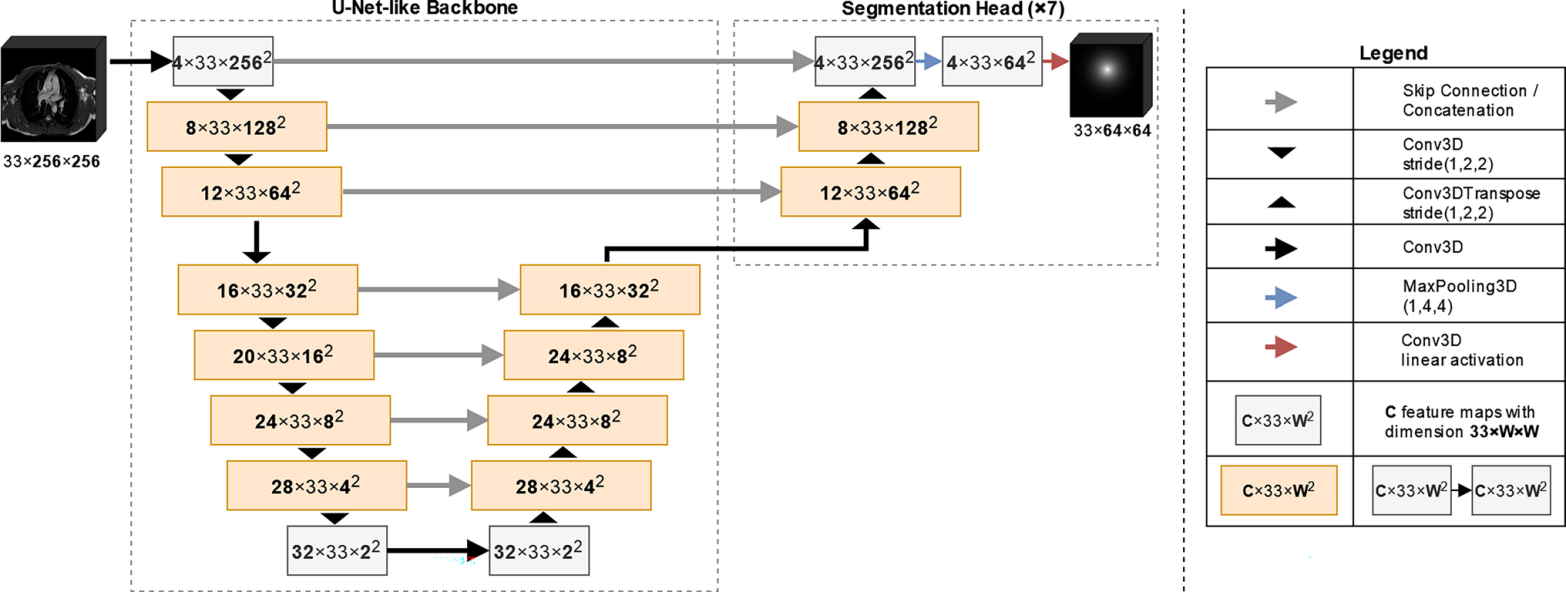
Illustration of the neural network architecture used for the detection of each of the seven landmarks. The network had a U-Net-like backbone and a segmentation head for each landmark. In the illustrated setting, splitting into segmentation heads was performed above depth 3

The architecture of the model was inspired by the U-Net [17] and is illustrated in Fig. 5. The network had a maximum depth of 7. Here and in the following, we refer to ‘depth’ as the number of downsampling and hence necessary upsampling steps. The segmentation model consisted of a shared U-net-like 3D-convolutional encoder backbone and 7 segmentation heads, each yielding a 3D-segmentation volume predicting the heatmap for one specific landmark, Fig. 5. The shared encoder takes the 3D voxel volume of size 33 × 256 × 256 as input. With each downsampling (upsampling) step, the x and y input resolution was halved (doubled) through the use of 3D convolution (transpose convolution) with stride 1 × 2 × 2. Furthermore, the number of kernels—and hence, resulting feature maps—was increased (decreased) in each downsampling (upsampling) step by 4. Between each two consecutive down- or upsampling steps, a 3D convolution layer without stride was inserted. As in U-Net, feature maps of the same size before down- and after upsampling were concatenated via skip connexions. During upsampling, the network was split into seven separate segmentation heads. Upsampling in each segmentation head functioned exactly the same as mentioned before. After upsampling to the original resolution, final MaxPooling and 3D convolution layer with linear activation function were added to each segmentation head to provide the final output of the seven segmentation volumes, each of size 33 × 64 × 64.

Kernel weights were initialised with He initialisation [18]. All convolution layers use the *Keras API *[12] ‘same’ padding, i.e. the input to each layer is padded with zeros if necessary. All convolution layers except the aforementioned final linear regression layer in each segmentation head use the ReLU [19] activation function.

### Segmentation network training

The segmentation network was trained on Nvidia GeForce GTX 1070 and an Intel Core i7-4790 K CPU with 4.00 GHz for 32 epochs, using a learning rate of 5 × 10^–4^, Adam optimizer [20] and a batch size of 2. Mean Squared Error (MSE) was used as the loss function. The number of filters in the first layer (between 3 and 9) and the depth of the segmentation heads (between 2 and 6) were parameters of the hyper-parameter search. Also, the use of data augmentation was tested.

### Heatmaps to point cloud conversion and centring

The position of maximum value in the predicted heatmap was used as the predicted landmark position. If this maximum value reached at more than one position, the predicted landmark position was determined from the heatmap H(i,j,k) as those positions yielding maximum score S with

$$S = \mathop \sum \limits_{i = x - 3}^{x + 3} \mathop \sum \limits_{j = y - 3}^{y + 3} \mathop \sum \limits_{k = z - 3}^{z + 3} H\left( {i,j,k} \right)$$

and spatial coordinates *i*, *j*, and *k*.

Real-world coordinates of the landmark positions (in mm, not voxel number index) were combined to a point cloud. Point clouds were standardised in preparation for their use as input for the SVN by centring the point clouds to the point cloud centre position ${m}_{i}$ which was defined by ${m}_{i}=\frac{{\sum }_{j=1}^{7}{l}_{ji}}{7}$ with *i* being the spatial coordinate index, *j* the landmark index and ${l}_{ji}$ the real-world positions of the landmark in mm. Relative landmark positions ${L}_{ji}$ were then represented by landmark positions relative to the centre position ${m}_{i}$ according to $L_{ji} = l_{ji} {-}m_{i}$.

### Configuration of the SVM

The SVM used for classifying based on point clouds had a linear kernel and was otherwise configured with the default values provided by the Scikit-learn software library [29].

### Segmentation model metrics

The accuracy error of the predicted landmark positions ${l}_{ji}$ was calculated as Euclidian distance to the ground truth landmark position g_j,i_, i. e. $E=\Vert {g}_{ji}-{l}_{ji}\Vert$ (in mm). This displacement was calculated for each landmark number $j$ and for the validation data sets $i$ of each fold. It was presented as an average over all n data sets: ${E}_{j}=\frac{{\sum }_{i=1}^{n}{E}_{ji}}{n}$. Interobserver variability was represented as ${E}_{interj}=\frac{{\sum }_{i=1}^{n}\Vert {g}_{ji}-{h}_{ji}\Vert }{n}$, where h_ji_ is the landmark position annotated by a second observer. The comparison between network error and interobserver disagreement was quantified as ratio $\frac{{E}_{j}}{{E}_{interj}}$. Also, standard deviation for all landmarks as well as Bland–Altman Plots for each spatial component $x$,$y$,$z$ of the landmark prediction was determined.

### SVM metrics

The performance of the SVM was measured by accuracy defined as the fraction of correct classifications.

### Training of the SVM

Training of the SVM was based on the centred point clouds of the data set using random initial weights. The SVM was trained anew on the training data for each cross-validation fold with and without preceding data augmentation.

### Direct classification

The classification accuracy of the proposed method was compared with the accuracy of a direct classification which carries the risk of being influenced by technical image features. Splitting of data and cross-validation were performed in the same way as for the proposed method.

## Results

**Fig. 6:**
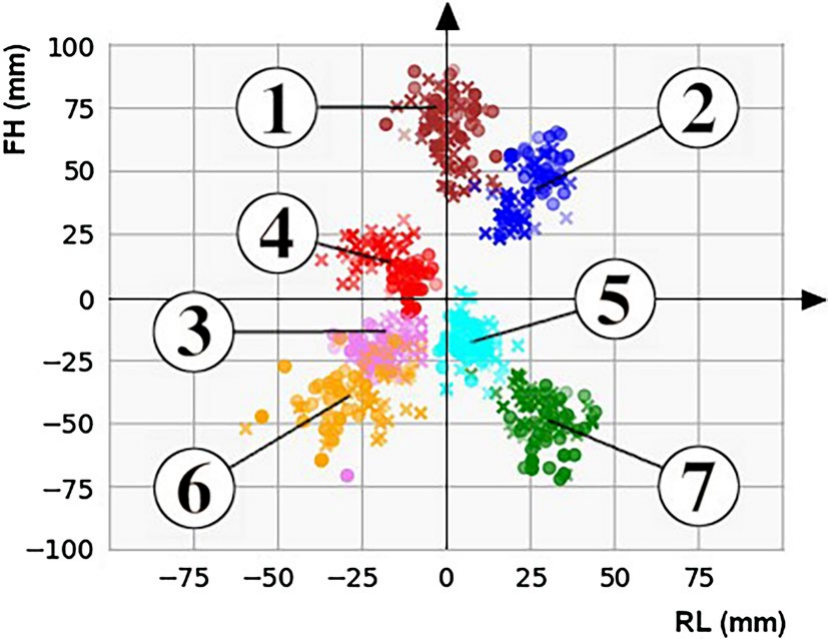
Distribution of annotated landmarks for patients (X) and healthy controls (●) after centring. Coordinates in right–left (RL) and foot–head (FH) directions are shown. Landmark numbers and their corresponding anatomical structures are defined Fig. 1

Distributions of annotated landmarks after centring are depicted in Fig. 6.

Training of one segmentation network took 20 min. The final neural network architecture had 6 filters in the first layer and applied a splitting into segmentation heads above depth 3. No augmentation was used for training.

**Fig. 7:**
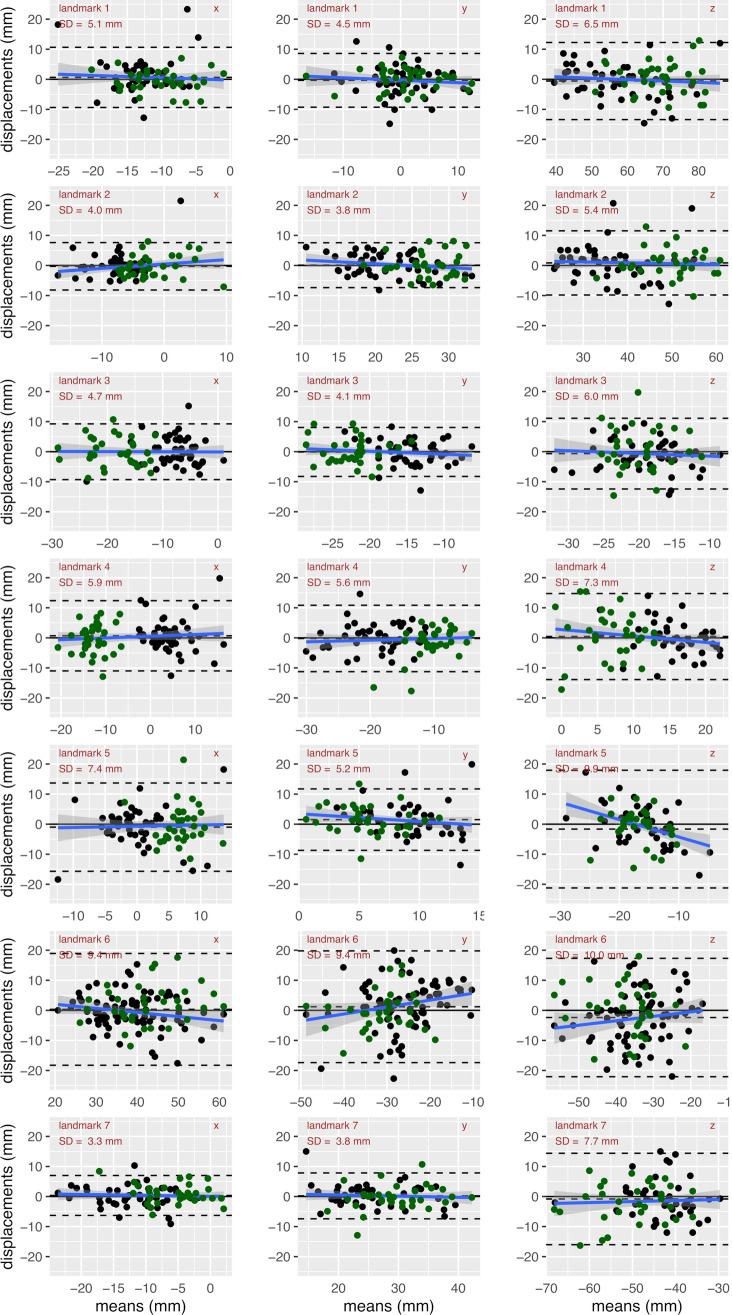
Bland–Altman analysis of the detected landmark positions in comparison with the annotation for the seven landmarks (rows: landmarks 1–7) and three spatial directions (columns: x, y, z). The dashed lines indicate the 95% confidence level. The blue line indicates the proportional bias fit. The standard deviation (SD) is stated in each plot. Black points represent HLHS patients, green points represent healthy controls

Average displacement and standard deviation between predicted and annotated landmarks as well as the interobserver variability for healthy controls, patients and the combined group are shown in Table 1. The Bland–Altman analysis for each spatial component is summarised in Fig. 7.

**Table 1:** Evaluation of landmark detection for healthy controls, HLHS patients and combined data

Landmark	Healthy controls (n = 33)			HLHS patients (n = 46)			Combined group (n = 79)		
	Network			Network			Network		
	Average displacement ± standard deviation		Interobserver variability	Average displacement ± standard deviation		Interobserver variability	Average displacement ± standard deviation		Interobserver variability
	(mm)	Relative	(mm)	(mm)	Relative	(mm)	(mm)	Relative	(mm)
1	0.6 ± 7.2	0.1 ± 1.0	± 7.0	2.0 ± 10.6	0.3 ± 1.5	± 6.5	1.0 ± 9.4	0.1 ± 1.3	± 7.3
2	1.0 ± 6.9	0.2 ± 1.3	± 5.4	0.7 ± 8.2	0.1 ± 1.5	± 4.4	0.8 ± 7.7	0.2 ± 1.4	± 5.1
3	0.5 ± 9.4	0.1 ± 1.3	± 7.0	1.5 ± 8.0	0.2 ± 1.1	± 5.0	0.7 ± 8.7	0.1 ± 1.2	± 7.0
4	1.5 ± 10.5	0.2 ± 1.0	± 10.1	1.4 ± 11.1	0.1 ± 1.1	± 4.2	0.8 ± 10.9	0.1 ± 1.1	± 8.0
5	2.7 ± 12.2	0.3 ± 1.4	± 8.8	2.4 ± 14.4	0.3 ± 1.6	± 7.7	2.5 ± 13.5	0.3 ± 1.5	± 8.6
6	2.3 ± 15.1	0.3 ± 1.8	± 8.4	5.7 ± 17.0	0.7 ± 2.0	± 9.0	2.7 ± 16.7	0.3 ± 2.0	± 8.7
7	1.1 ± 10.6	0.1 ± 0.7	± 16.0	1.4 ± 8.1	0.1 ± 0.5	± 10.4	1.0 ± 9.2	0.1 ± 1.6	± 16.5

Segmentation results are presented as average displacement from annotated landmark positions, standard deviations in units of mm as well as in relative units as ratio to the interobserver variability. Mean and standard deviation of the displacements are listed as average magnitude over four cross-validation folds in this table. The average displacement refers to the systematic shift in a certain spatial direction between the detected landmark and the landmark set the first observer, whereas the standard deviation refers to the scattering of the displacement. Landmark numbers and their corresponding anatomical structures are defined in Fig. 1

The following statements and the main findings are valid in each of these three groups, whereas numbers given in brackets are related to the combined group.

The average displacements were far below the standard deviations for all landmarks, which reflects the fact that the detected landmarks did not significantly deviate from the landmarks set by observer 1 in any particular preferred direction. Finding the best slice for setting the landmark was often ambiguous according to the observers and contributed to corresponding standard deviations.

The largest displacements and the standard deviations reached for landmarks 6 (apex, displacement 2.7 mm, standard deviation 16.7 mm) and 5 (mitral valve, displacement 2.5 mm, standard deviation 13.5 mm). For other landmarks, average displacements were typically in the order of 1 mm and standard deviations were below 11 mm. Also, in comparison with the interobserver variability, the detection of the right ventricular apex (landmark 6) had the highest deviations (2.0 times higher than the interobserver variability). Deviations of all other landmarks had about the size of the interobserver variability (ratios ranging between 1.1 and 1.6).

It is striking that for landmark 7 (descending aorta at the level of the diaphragm), the standard deviation of the off-set between prediction and observer 1 was smaller than the inter-observer-variability. We would like to point out that the network was trained with annotations prepared by observer 1, such that the network learned from the strategies of observer 1, whereas the interobserver variability represents differences between observer 1 and observer 2.

Training of the classifier on manual annotation training data took less than 1 min.

When skipping the landmark detection and using manual annotation test data directly as input to classifier prediction, the classification accuracy was 100.0%. All datasets were correctly classified. After landmark detection using the final hyper-parameters, the average classification accuracy was 98.7%. All but one dataset was correctly classified. Coefficients learned by the SVM are listed in Table 2 in terms of maximum and root mean square (RMS) of the three spatial coordinates. The coefficients for the aortic valve landmark had the largest values followed by the tricuspid valve.

**Table 2:** Classifier weights for the anatomical landmarks

Landmark	Classifier coefficients	
	Max	RMS
1 Origin of the left common carotid artery	0.003	0.016
2 Aortic isthmus	– 0.008	0.035
3 Tricuspid valve	0.046	0.053
4 Neo-aortic valve / aortic valve	**0.053**	**0.084**
5 Mitral valve	0.009	0.023
6 Right ventricular apex	0.006	0.020
7 Desc. aorta at diaphragm level	0.019	0.023

Landmark 4 contributes to classification with the largest classifier coefficients, see values highlighted in bold

Classifier weights are listed as maximum and root mean square (RMS) of the three spatial components. Presented results are averages obtained with the four cross-validation folds

Changing the number of filters in the first layer from 6 to 3 (9) changed the classification accuracy to 96.2% (89.9%). The variation in the depth of the segmentation heads from 4 down to 2 or up to 6 had no effect on the classification accuracy of 98.7%. Using data augmentation for training the neural network had no further effect on the classification accuracy. Also, augmentation of point clouds used for classification had no further effect on the accuracy.

The accuracy was about as high as the classification accuracy of a direct classification (98.8%) which carries the risk of being influenced by technical image features.

## Discussion

The prospect of being able to automatically gain information relevant for CMR image analysis opens up new potential to assist the evaluating physician [21, 22].

At the time of the CMR scan, the diagnosis of HLHS is already known. However, automated analysis of the cardiovascular anatomy can lead to important improvements of analysis software. The information from such automated analysis could be used to quickly jump to anatomic positions in the images and guide the physician more efficiently through the analysis depending on the detected condition, which may ultimately improve work flow and save analysis time. This CMR study used a deep neural network to detect anatomical landmarks and demonstrated their potential use for identification of HLHS. The approach could serve as a model for detecting certain cardiac anatomies in complex congenital heart defects. For this study, patients with HLHS after Fontan completion with a TCPC were included. However, since TCPC or upper cavopulmonary connexion was not a defined landmark, the method could be suitable for all HLHS patients after Norwood operation.

Based on the high accuracy of 100.0% for classification of manual annotation test data, the concept to classify HLHS patients and healthy controls by the spatial arrangement of anatomic landmarks was proven to be feasible. The tailor-made neural network in combination with the use of a linear SVM classifier turned out to be suitable to distinguish HLHS patients from healthy controls with an excellent overall classification accuracy of 98.7%.

Regarding the fact that differences in the detected landmarks had no impact on the classification accuracy for several hyper-parameters such as the depth of the segmentation heads or the use of augmentation, we conclude that the total performance of the method is robust with respect to these hyper-parameters.

Training of the SVM determined the largest classifier weights for the aortic valve landmark, which is the landmark that is shifted between healthy controls (native aortic valve) and patients (neo-aortic valve), as reflected by the displacement of red points and crosses in Fig. 6.

The development of a tailor-made neural network with a shared U-net-like 3D-convolutional encoder backbone and a segmentation head for each landmark was demonstrated to be a suitable use case. The backbone was trained with all landmarks. This led to improved performance compared to preliminary study results which used a separate neural network for each landmark. The reduced number of parameters also led to reduced computation cost. The actual classification was performed after landmark detection and based exclusively on the landmark positions relative to each other.

Using separate decoding heads for the detection of landmarks instead of a multichannel output has the advantage that particular decoding heads can be trained separately without affecting the performance for the detection. However, the use of separate decoding heads is not obligatory.

The challenges regarding explainability and reliability of neural networks for medical tasks have been noted in recent studies [23–25]. Clinical imaging data valuable for training neural networks are often available retrospectively. Retrospective data carries the risk that classification may be influenced by scan parameters, either because the data sets for the groups stem from different centres or because in a single centre, scan protocols and parameters are specifically used for a certain diagnosis or group. In direct classification, it often remains unclear which of the features found in the neural network represent clinically meaningful properties [26]. Using deep neural networks for clinical processes is therefore controversial [2]. It has been pointed out that heatmaps may be used to ensure models are capturing valid image signatures to further expand clinical usefulness [2].

As demonstrated in this study, decoupling the identification of clinically meaningful anatomic landmarks obtained using heatmaps from the actual classification can be valuable to improve transparency of classification. Landmarks were verified (Fig. 6; Table 1) and the impact of the landmarks on the result was analysed (Table 2), whilst a bias of undesired decision markers in the classification was prevented. Inappropriate decision markers such as CMR scan parameters had no direct influence on the classification. Due to the bias of inappropriate decision markers, a direct classification can achieve increased values for accuracy. Nevertheless, it was demonstrated that the classification accuracy of the proposed method was even about as high as the classification accuracy for direct classification whilst the decision was strictly based on clinically meaningful parameters.

Using heatmaps, the network included a larger image context in the identification of landmarks than using a direct regression of landmarks. This method has been recommended due to its robustness [14–16].

Different anatomical variations and different surgical procedures may increase inhomogeneity of imaging data sets from patients with complex congenital heart defects. Data inhomogeneity in conjunction with a small number of cases makes a classification challenging. Since the proposed method allows us to include healthy control data for training, it is only weakly limited by the number of patients with congenital heart disease and may, for instance, be applicable to the classification of HLHS subgroups with relatively small sample sizes in the future studies.

A limitation of this study was the small group size of 46 HLHS patients and 33 healthy controls. However, a high classification accuracy of 98.7% was achieved. In order to avoid biases in our training and validation data due to the small dataset size, we performed k-fold cross-validation [27, 28] for model selection and evaluation purposes.

Furthermore, in the current approach, neural network training applied equally to all heads. The mean squared error loss function was found to reach a saturation for the landmarks at different epochs. Future work may consider to first train the network backbone, freeze it and then train the heads separately with variable hyper-parameters.

This study presents an innovative method for machine-learning-based identification of HLHS patients after TCPC completion using CMR datasets based on the geometric arrangement of anatomical landmarks. The classification accuracy of the full method was 98.7%, whereas the final step of classification had an accuracy of 100.0% when using manually annotated landmarks. The automatic identification of the cardiovascular anatomy can be an important improvement of analysis software in the future, which may ultimately improve work flow and save analysis time.

## Data Availability

The numerical results of this study are available on request from the corresponding author (DDG).

## Abbreviations

HLHS: Hypoplastic left heart syndrome
TCPC: Total cavopulmonary connexion
SSFP: Steady-state free precession
CNN: Convolutional neural network
SVM: Support vector machine
